# Phenocopy in a patient with triple negative breast cancer: a case report

**DOI:** 10.31744/einstein_journal/2023RC0319

**Published:** 2023-11-10

**Authors:** Gustavo Henrique Morcelli da Costa, Eduarda Scoto Dias, Naiara Bozza Pegoraro, Camila Nemetz Kohler, Salmo Raskin, Liya Regina Mikami

**Affiliations:** 1 Faculdade Evangélica Mackenzie do Paraná Curitiba PR Brazil Faculdade Evangélica Mackenzie do Paraná , Curitiba , PR , Brazil .; 2 Centro de Aconselhamento e Laboratório Genetika Curitiba PR Brazil Centro de Aconselhamento e Laboratório Genetika , Curitiba , PR , Brazil .

**Keywords:** Phenotype, Breast neoplasms, Mutation, Genetic predisposition to disease, Genes *BRCA1*, Genes *BRCA2*

## Abstract

A total of 1.67 million breast cancer cases per year are reported worldwide. Of these, 5%–10% are caused by inherited mutations. Phenocopy is a rare phenomenon, with only a few cases reported in the literature. In phenocopies, phenotypes identical to those with genetic origin occur because of environmental factors rather than familial mutations. We describe a case of phenocopy in a 44-year-old female patient with triple-negative breast cancer. The mother and sister wee heterozygous for c.1813delA, p.Ile605TyrfsTer9 in *BRCA2* . The patient underwent genetic testing for *BRCA1* and *BRCA2* and exome sequencing. Familial or other cancer variants were not detected. The most accepted phenocopy theory is that patients without genetic variants but who are carriers of these mutations undergo cellular changes due to environmental factors, increasing the risk of breast cancer. Therefore, the detection of phenocopy in patients with breast cancer is important in clinical practice.

## INTRODUCTION

Breast cancer is considered the most common malignancy in women and causes approximately 1.67 million new cases per year worldwide. ^( 1 )^ In Brazil, approximately 66,280 new cases were reported in 2022. According to the data from the *Instituto Nacional do Câncer* , this represents an adjusted incidence rate of 43.74 cases per 100,000 women, which makes breast cancer the number one cause of cancer mortality in the female population. ^( 2 )^

Symptoms are only observed in patients with more advanced stages. These include the formation of a palpable lump with an irregular contour that is usually painless, skin retraction, inversion of the mammary papilla, bloody nipple discharge, breast skin edema, and axillary lymph node enlargement. ^( 3 )^

The patient’s family history is crucial to verify disease recurrence, especially in first-degree relatives, such as grandmothers, mothers, aunts, and sisters. Patients with breast and/or ovarian cancer segregated in the family, mainly in first-degree relatives, should be examined for the presence of distinct manifestations such as breast pain, sudden weight loss, bone pain, tiredness, and nipple discharge. Therefore, these patients should be screened continuously. ^( 4 )^

Several histological types of breast cancer, including molecular variations, have been described. Initially, they were classified as invasive and noninvasive. Noninvasive tumors do not affect the surrounding tissues, with ductal carcinoma in situ and lobular carcinoma in situ being the most common types. In invasive tumors, cancer cells leave the breast tissue, pass through the blood and lymphatic vessels, and spread to different regions of the body, such as the brain and lungs. Some types of invasive cancers include infiltrating lobular, medullary, and triple-negative carcinomas. The development of benign or malignant tumors is stimulated by carcinogenic factors. ^( 5 )^

Several genes have been identified as etiological agents or predisposing factors in breast cancer development. Abnormal mutations and the amplification of oncogenic and anti-oncogenic factors play central roles in the initiation and development of tumors. The main regulatory agents in the oncogenesis process are the *BRCA1, BRCA2* , and *TP53* genes. ^( 6 )^

In some cases, individuals may present phenotypes identical to those genetically originating from mutations, mainly in the *BRCA1, BRCA2* , and *TP53* genes, but that are triggered exclusively by environmental factors without familial mutations. This phenomenon is called phenocopy. Rosa et al. reported a case of phenocopy in a patient with ductal in situ breast cancer who tested negative for mutations in the *BRCA1* and *BRCA2* genes. The patient in the aforementioned study, as well as that in the present study, had relatives diagnosed with the most diverse types of cancer. Breast, esophageal, prostate, and ovarian cancers were among the different cancer types. ^( 7 )^

The present study demonstrated the importance of increasing the awareness of physicians and society regarding the existence of phenocopies. An individual can be affected by tumors caused only by the effect of the environment in the human body, even in the absence of genetic variants associated with the condition. In addition, the patients should be informed that their descendants do not have an increased risk of developing a cancerous condition owing to the recurrence of the disease as a result of genetic predisposition, as they will not be carriers of such variants. Thus, this study aimed to describe a rare case of phenocopy in a patient with breast cancer and a *BRCA2* gene mutation in the family.

## CASE REPORT

The patient was diagnosed with breast cancer with a genetic variant of *BRCA2* segregated in the family. A retrospective evaluation of patients’ medical records was performed. Data were collected from the medical records and were provided by the family through anamnesis. The following variables were examined: history of the disease, family history of the disease and mutations, material used for diagnosis, and results of molecular tests.

The female patient was 44 years old, experienced menarche at the age of 14 years, was G2P2A0, and had maternal relatives diagnosed with cancer. At reproductive age, the patient had a palpable nodule noted on breast self-examination. She was later diagnosed with triple-negative breast cancer and was treated with quadrant chemotherapy owing to the precise location of the tumor. Immunohistochemical tests of breast tissues showed the absence of estrogen and progesterone receptors.

**Figure 1:**
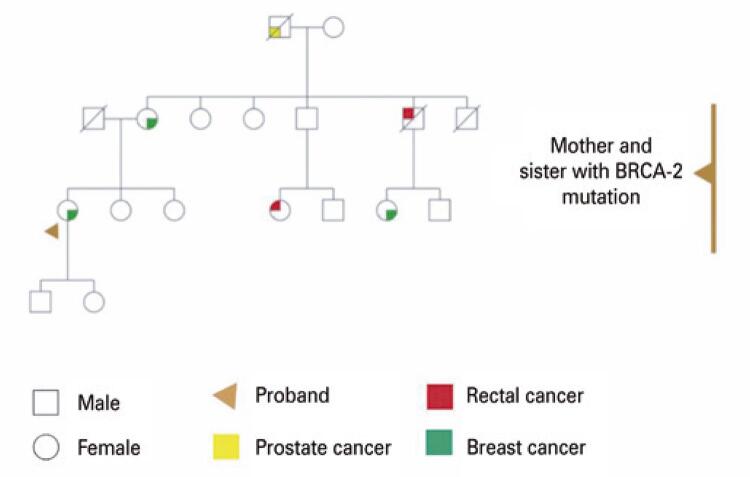
Family herdogram patient

Currently, the patient has two children, a 25-year-old boy and a 13-year-old girl. Genetic counseling in order to assess the risk of recurrence in her offspring, as her mother and younger sister wee heterozygous for the mutation c.1813delA, p.Ile605TyrfsTer9 in the *BRCA2* gene. The patient’s mother developed human epidermal growth factor receptor-positive breast cancer in the left breast at the age of 53 years. At 69 years of age, she developed a Breast Imaging Reporting and Data System category 4 lump in the right breast and underwent prophylactic surgery for tumor removal. The patient’s sister had the same genetic variant detected during the molecular testing of *BRCA1* and *BRCA2* genes at the age of 39 years and underwent preventive double mastectomy at the age of 42 years ( Figure 1 ).

The patient underwent two consecutive genetic tests: next-generation sequencing of *BRCA1* and *BRCA2* genes using peripheral blood samples and whole exome sequencing with analysis of copy number variation using saliva and peripheral blood samples to rule out the hypothesis of somatic mosaicism. Neither test showed the presence of pathogenic or likely pathogenic variants in genes associated with increased susceptibility to breast or ovarian cancer.

This study was approved by the Research Ethics Committee of the *Faculdades Integradas do Brasil/Complexo de Ensino Superior do Brasil* (CAAE: 35313414.7.0000.0095; # 777.566.

## DISCUSSION

Phenocopy occurs when an individual presents a phenotype identical to that caused by genetic factors; however, in these patients, the phenotype is caused exclusively by environmental factors that mimic the behavior of the genetically produced one. ^( 7 )^

Scientific publications regarding breast cancer have reported contrasting findings. Therefore, whether patients who are negative for genetic mutations but have first-degree relatives with breast cancer and genetic mutations have a higher likelihood of developing breast cancer than the general population remains unclear. ^( 8 )^

Bernholtz et al. analyzed the rate of breast cancer development in Jewish families. They concluded that there was no significant difference in the risk of disease development between individuals with genetic variants in the *BRCA1* and/or *BRCA2* genes and patients without these variants, but with first-degree relatives who inherited them. ^( 9 )^ In 2007, Goldgar et al. observed that the risk of developing cancer was two times higher in patients without the variants that segregated in their families than in the general population. ^( 10 )^ In a study by Evans et al., patients with first-degree relatives who had breast cancer and genetic variants in *BRCA1* or *BRCA2* and who tested negative for the same mutations were twice as likely to develop breast cancer compared with individuals of the same age in the general population. ^( 11 )^

The most accepted theory is that patients who are negative for genetic mutations but have relatives with *BRCA1* or *BRCA2* mutations have more genetic modifiers that can increase the penetrance of both *BRCA1* and *BRCA2* genes. In addition, individuals who are *BRCA2* mutation carriers are more likely to produce genetic modifiers. This leads to a greater likelihood of developing breast cancer, especially with advancing age, compared with individuals who are carriers of *BRCA1* mutations, which occurred in the patient in the present study. ^( 12 )^

## CONCLUSION

The patient in this study did not have pathogenic genetic variants but had relatives with a genetic mutation in *BRCA2* . She developed breast cancer with a different histological type from that of her mother. Therefore, this was a rare case of phenocopy. This findings from this report are highly significant in clinical practice because, in addition to highlighting a rare phenomenon that has not been extensively described in the literature, it corroborates the need for further studies to determine the likelihood of developing the disease in this patient group, owing to the inconsistency in the reports of published studies. Taking into consideration only the genetic variants associated with cancer risk, the probability of the descendants of these individuals to develop breast cancer is considerably lower than the general population. Therefore, the probability of disease recurrence in the offspring due to mutations in the *BRCA2* gene is practically zero.
